# The Influence of Temperature on the Photoelectric Properties of GeSe Nanowires

**DOI:** 10.3390/molecules29122860

**Published:** 2024-06-16

**Authors:** Qiaoping Liu, Zhiyong Zhang, Fuchun Zhang, Yanning Yang

**Affiliations:** 1 School of Information Science and Technology, Northwest University, Xi’an 710127, China; liuqiaoping1211@126.com; 2 School of Physics and Electronic Information, Yan’an University, Yan’an 716000, China; yadxzfc@yau.edu.cn (F.Z.); yadxyyn@163.com (Y.Y.)

**Keywords:** two-dimensional materials, GeSe nanowires, physical vapor deposition, substrate, photoelectric properties

## Abstract

Using physical vapor deposition (PVD) technology, GeSe nanowires were successfully fabricated by heating GeSe powder at temperatures of 500 °C, 530 °C, 560 °C, 590 °C, and 620 °C. The microstructure, crystal morphology, and chemical composition of the resulting materials were thoroughly analyzed employing methods like Scanning Electron Microscopy (SEM), X-ray Diffraction (XRD), plus Raman Spectroscopy. Through a series of photoelectric performance tests, it was discovered that the GeSe nanowires prepared at 560 °C exhibited superior properties. These nanowires not only possessed high crystalline quality but also featured uniform diameters, demonstrating excellent consistency. Under illumination at 780 nm, the GeSe nanowires prepared at this temperature showed higher dark current, photocurrent, and photoresponsivity compared to samples prepared at other temperatures. These results indicate that GeSe nanomaterials hold substantial potential in the field of photodetection. Particularly in the visible light spectrum, GeSe nanomaterials exhibit outstanding light absorption capabilities and photoresponse.

## 1. Introduction

In the context of an increasingly severe global energy situation, exploring and developing sustainable energy sources and highly efficient photoelectric devices is not only an inevitable trend in scientific and technological development but also key to meeting future societal needs. Particularly in the field of new energy, research on new materials with high-efficiency photoelectric conversion characteristics has become the frontier of scientific exploration. Among various research directions, two-dimensional substances have attracted significant interest from researchers globally and locally, owing to their distinct physical and chemical traits and their expansive potential in applications such as photoelectric conversion, sensors, and photocatalysis.

IV–VI Group Compounds, such as germanium selenide (GeSe), tin sulfide (SnS), and tin selenide (SnSe) [1], have become research hotspots in recent years due to their stable chemical properties, abundant availability, low cost, and non-toxic nature. Among them, two-dimensional GeSe nanomaterials have attracted particular attention due to their excellent photoelectric properties [2]. GeSe is a binary semiconductor material with a density of 5.56 g/cm^3^. Its crystal structure is orthorhombic, belonging to the space group Pnma 62, with lattice constants a = 1.084 nm, b = 0.383 nm, and c = 0.439 nm. GeSe represents a standard layered compound; Ge and Se atoms link through robust chemical bonds, whereas the layers unite via van der Waals forces. From a physicochemical perspective, GeSe possesses a unique crystal structure, exhibiting significant anisotropy. It has a molecular weight of 151.6 and a high melting point o 670 °C. Its direct bandgap is approximately 1.16 eV [3], with a high absorption coefficient above 10^5^ cm^−1^ [4], high carrier mobility (approximately 128.6 cm^2^V^−1^s^−1^), and a high dielectric constant (>15), which can effectively shield the effects of charged defects [5]. Additionally, its good carrier lifetime (approximately 9.9 ns [6]) and ease of sublimation [7] markedly augment its prospective uses in the realm of photo electronics. As such, it has become a focal point of research in photonic and infrared photodetection [8,9]. Therefore, in-depth research into the preparation and photoelectric properties of GeSe nanomaterials is crucial for the development of new generations of high-efficiency photoelectric devices.

In the discussion of the preparation techniques for GeSe materials, mechanical exfoliation, as a traditional and widely used method, involves the direct physical peeling of materials to obtain layered structures. Due to its straightforward and convenient operation, it played a significant role in the early research [10,11,12]. However, as research demands deepen and application fields continue to expand, a series of limitations of mechanical exfoliation in preparing GeSe have gradually emerged. These limitations primarily include the uncontrollability of the thickness, size, and cleanliness of the exfoliated samples, which significantly restrict the performance and reliability of the materials in precise scientific research and practical applications [13].

In light of this, researchers have continually explored and experimented with new synthesis methods in recent years to overcome the limitations of mechanical exfoliation, successfully synthesizing GeSe materials through various innovative approaches. These new methods aim to achieve higher sample quality and better controllability to accommodate the in-depth study of the performance and application potential of GeSe. These synthesis techniques encompass, though are not confined to, Physical Vapor Deposition (PVD) [14,15,16], Chemical Vapor Deposition (CVD) [17,18,19], and the solvothermal method [20].

Although significant advancements have been made in the laboratory preparation techniques for GeSe nanomaterials, in-depth studies on their preparation methods and photoelectric properties remain insufficient. The preparation of high-quality GeSe nanomaterials and the precise evaluation of their photoelectric performance are not only crucial for elucidating the mechanisms of their photoelectric responses but also foundational for their widespread adoption in practical applications such as photoelectric devices. Physical vapor deposition technology has the advantages of a simple process, no pollution, fewer consumables, uniform and dense film formation, and a strong binding force with the matrix. Therefore, the aim of this study is to prepare GeSe nanowires by physical vapor deposition and to analyze the impact of various temperatures (500 °C, 530 °C, 560 °C, 590 °C, and 620 °C) on their morphology, X-ray Diffraction (XRD) patterns, and photoelectric properties to explore their potential applications in the field of photoelectronics.

## 2. Results and Discussion

### 2.1. Structural Characterization

**Figure 1:**
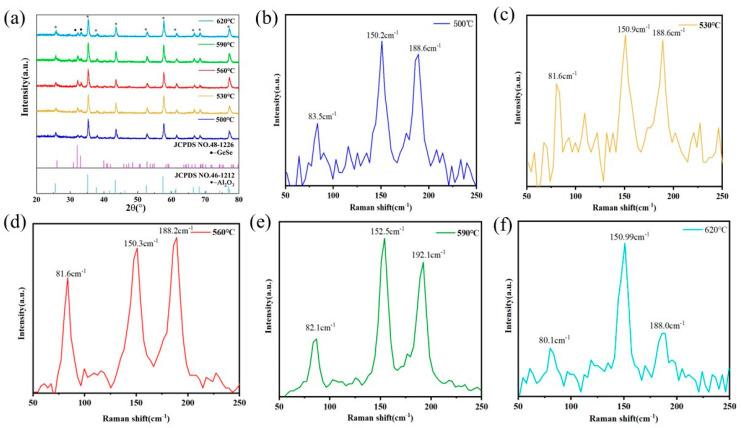
(**a**) XRD patterns of GeSe nanowires grown at various temperatures. (**b**–**f**) Raman spectrum of the GeSe nanowires grown at 500 °C, 530 °C, 560 °C, 590 °C, and 620 °C.

Figure 1a displays the X-ray diffraction (XRD) patterns of GeSe nanowires grown at different temperatures. In the spectra, the two prominent peaks marked by circles at 32.18° and 33.24° correspond respectively to the (111) and (400) planes of the orthorhombic phase of GeSe structure (according to JCPDS NO.48-1226, a = 10.84 Å, b = 3.834 Å, c = 4.39 Å) [21]. The peaks marked with asterisks represent the characteristic diffraction peaks of the substrate material–ceramic plates (Al_2_O_3_). Due to the high intensity of the diffraction peaks from the ceramic substrate, the characteristic peaks of GeSe are relatively weak. The analysis of the spectra reveals that as the heating temperature increases, the intensities of the (111) and (400) planes’ diffraction peaks first significantly increase and then decrease, indicating an initial improvement followed by a decline in the crystalline quality of the GeSe nanowires. The diffraction peaks reach their relative maximum intensities at a temperature of 560 °C, suggesting the highest relative crystalline quality of GeSe nanowires at this temperature. As the heating temperature continues to rise, the residual stress between the GeSe crystal nuclei adsorbed on the Al_2_O_3_ substrate and the substrate decreases, and the rate of GeSe growth along the energetically favorable direction of the nuclei increases, thus reducing the internal defects and enhancing its crystallinity. However, when the temperature reaches 590 °C, the rapid growth rate of the GeSe nanowire array leads to an increase in internal defects, resulting in decreased crystallinity. At 620 °C, the growth leads to nucleation and subsequent merging of nuclei, causing more internal defects and grain boundaries, thus further diminishing the crystallinity.

Figure 1b–f presents the Raman spectrum of GeSe nanowires grown at 500 °C, 530 °C, 560 °C, 590° C, and 620 °C. According to the symmetry principles of the D_2h_^16^ group, GeSe theoretically possesses 12 Raman active vibrational modes, including 4 A_g_ modes, 2 B_1g_ modes, 4 B_2g_ modes, and 2 B_3g_ modes [22,23,24]. In the Raman spectrum shown in Figure 1b–f, we successfully observed three main vibrational modes, located near 81 cm^−1^ (A_g3_ mode), 150 cm^−1^ (B_3g_ mode), and 188 cm^−1^ (A_g1_ mode). These results are highly consistent with previous research reports on the Raman peak positions of GeSe [25,26,27], thereby confirming the structure and composition of the synthesized GeSe nanowires.

**Figure 2:**
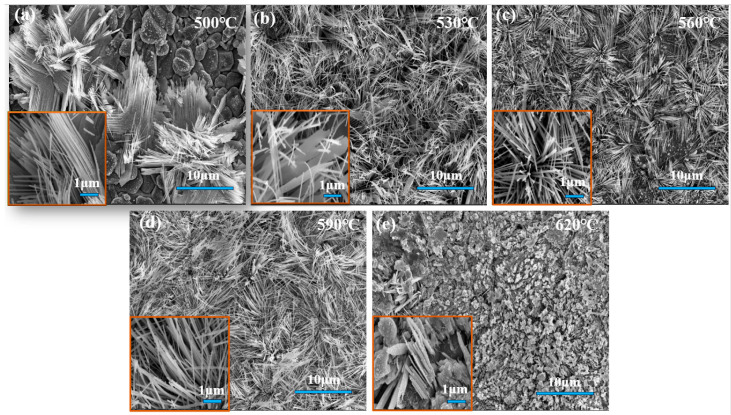
SEM images of GeSe nanowires grown at different temperatures: (**a**) 500 °C, (**b**) 530 °C, (**c**) 560 °C, (**d**) 590 °C, and (**e**) 620 °C.

Figure 2 depicts scanning electron microscope (SEM) images of GeSe nanowires synthesized at various temperatures. It is evident that under conditions of 500 °C (Figure 2a), the grown GeSe nanowires exhibit relatively sparse distribution, accompanied by uneven nanowire shapes, with some portions even revealing the underlying ceramic substrate. GeSe nanowires prepared at 530 °C (Figure 2b) are comparatively denser in arrangement yet display irregular features, featuring nanowires with varying diameters. In contrast, GeSe nanowires grown at 560 °C (Figure 2c) exhibit a sparser distribution with uniform diameters. Those synthesized at 590 °C (Figure 2d) are again relatively dense but with a disorderly arrangement, showing nanowires with more consistent diameters. Lastly, GeSe nanowires prepared at 620 °C (Figure 2e) display a granular structure.

From the perspective of crystal growth, in the process of preparing GeSe nanowires via physical vapor deposition (PVD)), the precursor GeSe evaporates and decomposes into gas-phase molecular groups at high temperatures, subsequently reaching the surface of an Al_2_O_3_ ceramic substrate through diffusion. Once the concentration reaches a critical value, nucleation spontaneously occurs at lower-energy positions on the substrate surface. During further growth, GeSe atoms initially adsorb onto the substrate surface, then preferentially bond at the lower-energy sites of the nuclei, causing the nuclei to gradually enlarge. Eventually, GeSe nanowires are formed on the Al_2_O_3_ ceramic substrate. Additionally, temperature also affects the adsorption and desorption processes of GeSe vapor on the substrate surface, thereby influencing the growth rate of GeSe. When the reaction temperature is 500 °C, the low concentration of vapor generated from the evaporation decomposition of GeSe powder results in fewer adsorbed and nucleated microcrystals on the Al_2_O_3_ ceramic substrate surface, leading to a lower density. During subsequent nucleation growth, GeSe grows slowly along the lower-energy direction, resulting in sparse and unevenly sized GeSe nanowires. However, at 530 °C, the increased concentration of vapor generated from the evaporation decomposition of GeSe powder leads to a higher density of adsorbed and nucleated microcrystals on the substrate surface. During nucleation growth, the growth rate of GeSe along the lower-energy direction increases, resulting in denser GeSe nanowires, albeit with uneven diameters. As the temperature further increases to 560 °C, the vapor generated from the evaporation decomposition of GeSe powder continues to increase in quantity, further raising the concentration. However, due to the excessively high temperature of the Al_2_O_3_ ceramic substrate surface, the probability of desorption of GeSe increases, resulting in a reduction in the number of nucleated microcrystals. During subsequent nucleation growth, the growth rate of GeSe along the lower-energy direction increases, leading to a decrease in the density of the GeSe nanowire array and a tendency towards uniform diameters. When the temperature continues to rise to 590 °C, the quantity of vapor generated from the evaporation decomposition of GeSe powder further increases, and the concentration continues to rise. However, with the increase in GeSe vapor concentration, desorbed GeSe molecular clusters rebound to the substrate surface after collisions and tend to re-adsorb at nucleation sites with stronger binding, resulting in a continued increase in the number and density of nucleated microcrystals on the substrate surface. During subsequent nucleation growth, the excessively rapid growth rate of GeSe along the lower-energy direction results in denser and irregularly shaped GeSe nanowires. When the temperature continues to rise to 620 °C, the quantity of vapor generated from the evaporation decomposition of GeSe powder becomes extremely large, with a very high concentration. The number of nucleated microcrystals rapidly increases on the surface of the Al_2_O_3_ ceramic substrate, resulting in very high density. During subsequent nucleation growth, the nuclei merge with each other, and smaller nuclei gradually disappear due to the reduction in surface free energy, leading to a rapid growth of GeSe crystals. Ultimately, irregularly shaped GeSe nanowires with larger dimensions are obtained [28].

**Figure 3:**
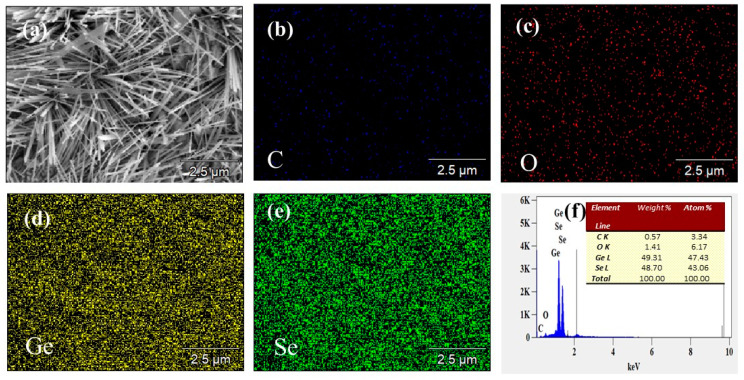
(**a**) SEM (**b**) C mapping (**c**) O mapping (**d**) Ge mapping (**e**) Se mapping (**f**) EDS (The inner illustration is the element distribution) of GeSe nanowires grown at 560 °C.

Through SEM-EDS and mapping analysis of GeSe nanowires prepared under conditions of 560 °C, we have obtained the elemental composition and distribution characteristics on the surface of the nanowires, as illustrated in Figure 3. The analysis reveals that the atomic percentages of germanium (Ge) and selenium (Se) in the sample are 47.43% and 43.06%, respectively. Additionally, traces of oxygen (O) and carbon (C) elements were detected, accounting for 6.17% and 3.34% of the composition, respectively. It is noteworthy that the atomic ratio of Ge to Se elements is close to 1:1, indicating a relatively balanced stoichiometry of GeSe nanowires. The detected oxygen and carbon elements are likely derived from air contamination or substrate materials, as no other foreign elements were found in the analysis of the sample.

Observation of the elemental distribution in the mapping images reveals that the distribution of germanium (Ge) and selenium (Se) elements in the sample is remarkably uniform and closely packed. This further confirms the uniformity and high purity of the synthesized nanowires. This result is consistent with the conclusions obtained from previous X-ray diffraction (XRD) and Raman spectroscopy analyses, all indicating that the obtained sample consists of high-quality GeSe nanowires.

**Figure 4:**
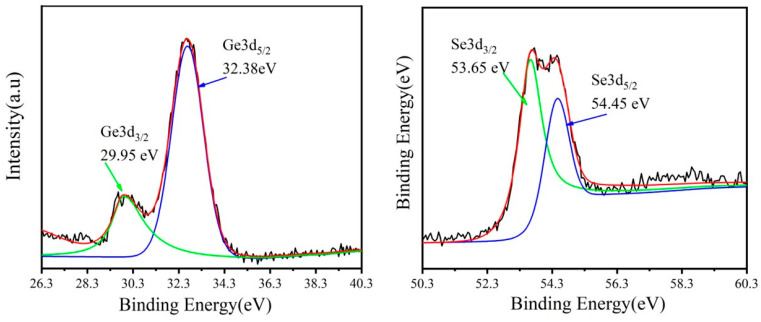
XPS spectra of (**a**) Ge 3d and (**b**) Se 3d in GeSe thin film. The black is the raw data, the red line is the fitting bus, and the green and red lines are sub-peaks.

In order to thoroughly investigate the chemical composition of the prepared germanium selenide (GeSe) nanosheets, this study employed X-ray photoelectron spectroscopy (XPS) to perform detailed characterization of the samples. The XPS spectra analysis results are shown in Figure 4. Figure 4a displays the XPS spectrum of Ge3d, revealing two prominent peaks located at approximately 29.95 eV and 32.28 eV, corresponding to the energy levels of Ge3d_3/2_ and Ge3d_5/2_ electron orbitals in the GeSe molecule, respectively. Furthermore, Figure 4b illustrates the XPS spectrum of Se3d, where two characteristic peaks are observed around 53.65 eV and 54.45 eV, representing the energy levels of Se3d_3/2_ and Se3d_5/2_ electron orbitals, respectively. By performing a fitting analysis of the binding energies of Ge and Se, the obtained results closely match the standard binding energy data reported in the literature for GeSe [29,30], confirming the high purity of the sample as GeSe, with no presence of any oxidation states observed in the sample.

**Figure 5:**
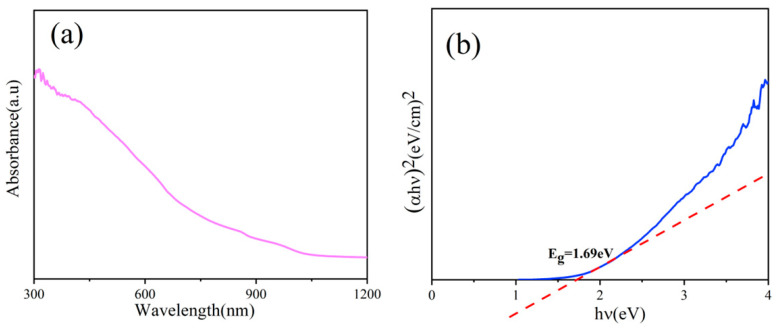
(**a**) UV-VIS absorption spectra and (**b**) optical band gap spectra of GeSe nanowires prepared at 560 °C.

The optical properties of GeSe nanowires were characterized by UV-Vis-NIR spectroscopy. The absorbance begins to decline sharply at about 450 nm and gradually decreases at 1050 nm (Figure 5a), indicating that GeSe nanowires have good light absorption potential. The band gap width of GeSe nanowires can be calculated by Tauc’s equation [31], which is shown as follows:

(ahv)^2^ = A (hv − Eg)

where a, h, v and A are absorption coefficient, Planck constant, photon frequency and constant respectively, and the linear fitting line with the *X*-axis gives a direct band-gap of 1.69 eV (Figure 5b), which is in good agreement with the previously reported results [11].

### 2.2. Computational Analysis

**Figure 6:**
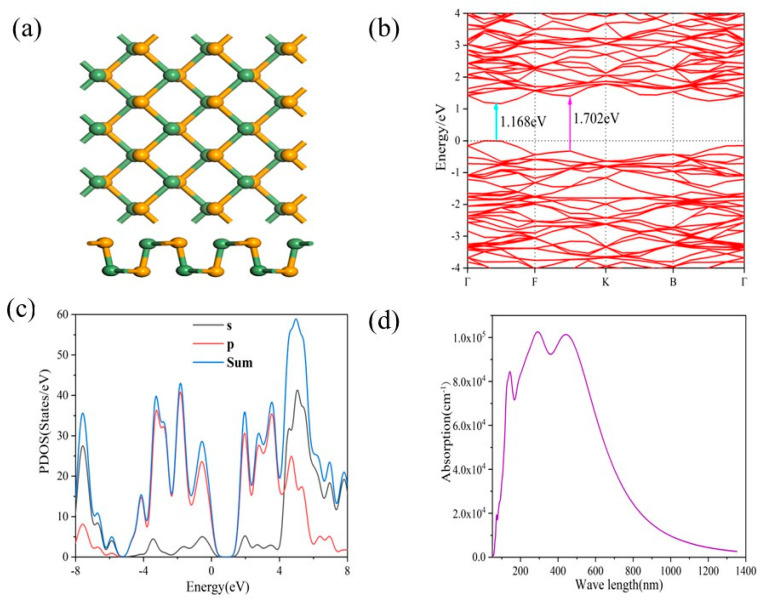
(**a**) The top and side views of the atomic structure model of GeSe. (**b**) Band structure of GeSe. (**c**) Partial density of states (PDOS) of GeSe. (**d**) Optical absorption of GeSe along the zigzag direction.

In this study, first-principles calculations were employed to systematically analyze monolayer GeSe, obtaining its band structure, partial density of states (PDOS), and optical absorption properties. The top and side views of the monolayer GeSe model are depicted in Figure 6a. By calculating the electronic band structure of single-layer GeSe using the GGA method, we predict that the possible direct transitions come from the direct band gaps of 1.168 and 1.702 in the Gamma-F and F-K directions, which are located in the visible and near-infrared spectral ranges around 737 nm and 1034 nm, respectively, as shown in Figure 6b. This characteristic renders GeSe highly promising for applications in photodetection.

In further analysis, Figure 6c illustrates the partial density of states (PDOS) of GeSe, revealing that the peaks near the Fermi level associated with P orbitals are significantly higher than those of S orbitals, indicating the dominant role of P orbitals in the electronic structure. Additionally, as shown in Figure 6d, theoretical calculations of optical absorption for monolayer GeSe cover a broad spectral range from ultraviolet to visible and even near-infrared regions. Particularly in the visible light region, the absorption intensity exceeds the magnitude of 10^5^. These computational results not only demonstrate the excellent performance of GeSe materials in broad-spectrum light absorption but also anticipate their vast potential applications in fields such as photodetection.

### 2.3. Photoelectric Performance Analysis

**Figure 7:**
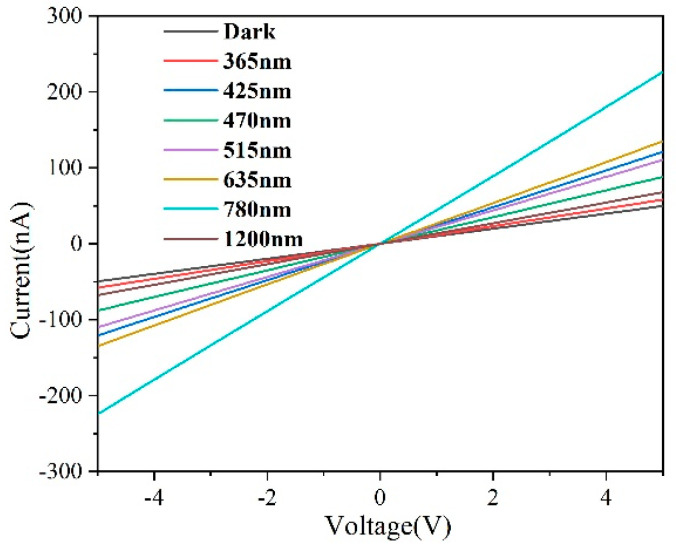
Photo response of GeSe nanowires grown at 560 °C under different wavelength light.

In order to investigate the photoresponse performance of GeSe photodetector devices under different wavelengths of light, the results obtained under laser irradiation at seven different wavelengths of 365 nm, 425 nm, 470 nm, 515 nm, 635 nm, 780 nm, and 1200 nm were compared with the calculated optical absorption spectra as references. The obtained results, as shown in Figure 7, indicate a good linear relationship between the current (I) and voltage (V), suggesting ohmic contact between the Ag metal and GeSe semiconductor [32,33]. Additionally, the photoelectric current generated by the constructed GeSe photo detector devices under different light sources exceeds their dark current, indicating responsiveness to various light sources. However, as the wavelength of the light source increases, the photoelectric current response of the GeSe photodetector devices initially increases and then decreases. The best photoelectric current response of the GeSe photodetector device is observed when the light source wavelength is 780 nm.

**Figure 8:**
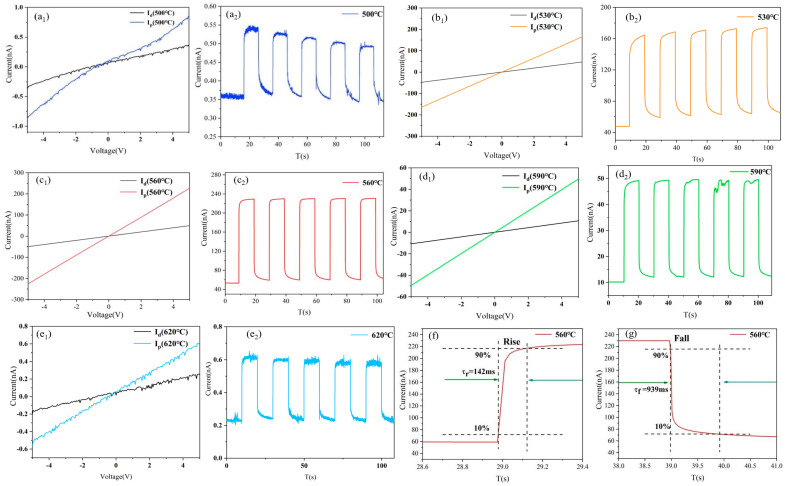
(**a_1_–e_1_**) and (**a_2_–e_2_**) respectively depict the I-V curves and photoresponse of GeSe nanowires prepared at 500 °C, 530 °C, 560 °C, 590 °C, and 620 °C under 780 nm light. (**f**) The green arrow in the figure marks the rising period, and the green arrow in the figure (**g**) marks the falling period respectively, of GeSe nanowires grown at 560 °C under 780 nm light.

In order to investigate the photoresponse of GeSe photodetector devices prepared at different temperatures under 780 nm wavelength light, their I-V curves, I-T curves, and response times are shown in Figure 8. From Figure 8 a_1_–e_1_, it can be observed that the dark current and voltage of the prepared GeSe photodetector devices exhibit a linear relationship, demonstrating good ohmic characteristics. Under illumination with 780 nm light, the photoelectric current of the prepared GeSe photodetector devices increases with increasing voltage, indicating a significant photoelectric response. Figure 8 a_2_–e_2_ displays the response characteristics of the different GeSe photodetector devices under a bias voltage of 5 V, with the current responding to the switching of light illumination over time.

R = (I_ph_ − I_d_)/(P_0_A), where R signifies the photoresponsivity, indicative of the photoelectric current generated per unit power of incident light at a designated wavelength across the device area [34,35]. In this context, I_ph_ represents the photocurrent under laser exposure, I_d_ denotes the dark current, A is the illuminated region of the GeSe device microstructure (−0.01 cm^2^), and P_0_ is the optical power density, recorded at 9.94 mW/cm^2^ [36,37]. The dark currents of different GeSe photodetector devices prepared at 500 °C, 530 °C, 560 °C, 590 °C, and 620 °C are 0.350 nA, 47.5 nA, 53.45 nA, 10.15 nA, and 0.232 nA, respectively. The photocurrents are 0.86 nA, 164.16 nA, 230.08 nA, 49.35 nA, and 0.627 nA, respectively. The sensitivity of the photodetector prepared at 560 °C and 590 °C is 4.3 and 4.86 respectively, which is better than the parameters of 2.59, 2.63 and 3.45 in the literature [33]. By calculation, the photoresponsivities of the photodetector prepared at 530 °C, 560 °C and 590 °C are determined as 11.74, 17.77, 3.94 (×10^−4^ A/W), respectively. All of them are higher than the response of 43.6–76.3 μA/W in reference [34].

**Figure 9:**
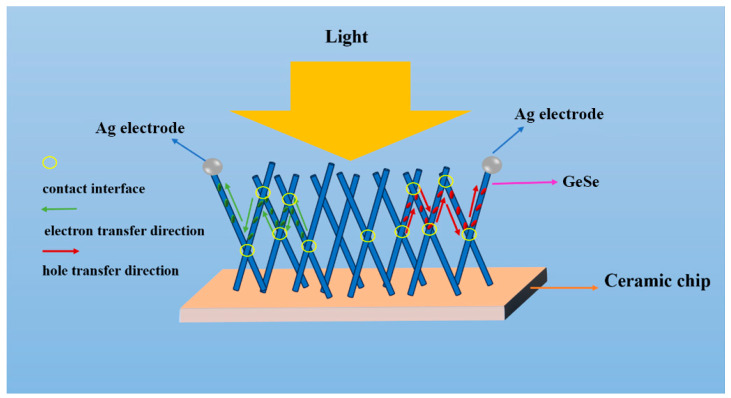
Principle of the GeSe nanowire photodetector.

When the temperature is 500 °C, the prepared GeSenanowires material is relatively sparse and does not completely cover the entire substrate surface, resulting in small dark and photocurrents, thus yielding a lower responsivity of the device. In our designed optoelectronic test system, the GeSe nanobelt device forms a “conductive network” where all individual nanowires contact each other for charge carrier transport, as shown in Figure 9. For a given area of the conductive network, the high density of the nanowires arrangement increases the direct contact area between nanowires, resulting in an increase in interface states and potential barriers and consequently lowering the dark current due to poor crystal quality. Conversely, a low nanobelt density reduces the contact area within the conductive network, resulting in fewer interface states and potential barriers, and thus increasing the dark current due to better crystal quality. Under illumination, the dense nanobelt arrangement with poor crystal quality impedes the transfer of photo-generated charge carriers through the lattice and interfaces, resulting in a smaller photocurrent. Conversely, a sparse nanobelt arrangement with better crystal quality facilitates the transfer of photo-generated charge carriers through the lattice and interfaces, leading to a larger photocurrent.

Compared to GeSe nanowires heated at 530 and 590 °C, those heated at 560 °C exhibit a relatively sparse arrangement with better crystal quality, resulting in a reduction in interface states and potential barriers within the conductive network, thereby yielding the highest dark current. Under illumination with 780 nm light, the GeSe nanowires with better crystal quality allow rapid migration of photo-generated charge carriers through the lattice and fewer migrations between nanorods, resulting in the highest photocurrent and, consequently, the highest responsivity of the photodetector device. Conversely, samples prepared under heating conditions of 620 °C consist of large irregular GeSe particles with poor crystal quality, leading to smaller dark and photocurrents and, consequently, relatively lower responsivity.

Figure 8f,g illustrates the light response of the device under 780 nm illumination (LPD = 9.94 mW/cm^2^). The rise time of the light response (τ_r_) is characterized as the duration for the photocurrent to ascend from 10% to 90%, while the fall time (τ_f_) is the period for the photocurrent to decline from 90% to 10%. The GeSe photodetector device manifests a rise time of 142 ms and a fall time of 939 ms at a 780 nm wavelength, indicating a stable and swift response. The rise time is lower than the times of 200 ms, 280 ms, and 150 ms in reference [4,13,38].

## 3. Experimental Section and Theoretical Calculations

### 3.1. Synthesis of Nanowires

**Figure 10:**
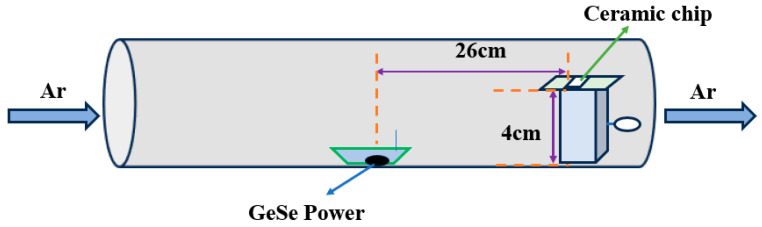
Schematic of the PVD process for synthesizing GeSe nanowires.

GeSe nanowires were successfully synthesized using the Physical Vapor Deposition (PVD) method, as depicted in Figure 10. The procedure commenced with the precise weighing of 25 mg of GeSe powder (160 mesh, 99.99% purity, supplied by Jiangxi Ketai New Materials Co., Ltd., Nanchang, China) placed in a 10 cm quartz boat at the center of a quartz tube. A ceramic piece, measuring 5 mm by 10 mm by 0.5 mm, was selected as the substrate for the experiment. Prior to the deposition of the GeSe film, the ceramic substrate was subjected to cleaning with a mixture of ethanol and acetone under ultrasonic agitation for approximately 30 min. After cleaning, the substrate is located downstream of the heating zone at a horizontal distance of 26 cm from the center of the GeSe powder source, and the vertical distance between the substrate and the bottom of the quartz tube is about 4 cm.

To thoroughly eliminate any air from the system, the interior of the quartz tube was evacuated using a vacuum pump. Subsequently, the tube was flushed with argon gas (Ar) at a flow rate of 450 sccm for 10 min, with the gas flow precisely controlled by a mass flow controller. Next, the temperature of the heating zone was adjusted to various set points (500 °C, 530 °C, 560 °C, 590 °C, and 620 °C) and maintained for 70 min at each temperature. Concurrently, the argon gas flow was adjusted to 190 sccm to guide the gas into the reaction tube. Once the reaction concluded, the apparatus naturally settled to ambient temperature. The ceramic substrate was then removed, revealing a uniformly grown black film on its surface. Most of the GeSe precursor powder in the quartz boat had reacted, leaving only a small amount of silvery-gray residue.

### 3.2. Fabrication of Ag Electrodes

The silver electrodes were prepared using a dotting technique. Specifically, a toothpick was used to dip into type 3701 silver paste, which was then evenly applied to the surface of the pre-prepared GeSe nanowires. The distance between the two electrodes was maintained at approximately 1 mm, ensuring an effective contact area of 0.01 cm^2^.

### 3.3. Characterization and Testing

The crystal structure of the samples was extensively analyzed using X-ray Powder Diffraction (XRD) to determine their phase composition. Additionally, the molecular vibration modes of the samples were investigated using a Raman spectrometer with an excitation wavelength of 532 nm, further verifying their structural information. Scanning Electron Microscopy (SEM) was utilized to examine the surface morphology and microstructure of the substances. X-ray Photoelectron Spectroscopy (XPS) was applied to analyze the chemical composition and electronic states on the surfaces of the materials. Simultaneously, Energy Dispersive X-ray Spectroscopy (EDS) served as a complementary technique, providing qualitative and quantitative information on the distribution and content of elements within the materials.

### 3.4. Performance Testing of Photodetectors

In this study, a Keysight B2901A (Shenzhen Zhongrui Yike Electronics Co., Ltd., Shenzhen, China) source meter was utilized as an external power source to measure the photoelectric performance of GeSe nanowires under various conditions. The experiment employed lasers of seven different wavelengths: 365 nm, 425 nm, 470 nm, 515 nm, 635 nm, 780 nm, and 1200 nm, to conduct detailed testing and analysis on GeSe samples prepared at different temperatures (500 °C, 530 °C, 560 °C, 590 °C, and 620 °C). A DHC GCI-7103M-B shutter (Daheng New Era Technology Co., LTD., Beijing, China) was used to switch the on/off states to determine the time response characteristics of the device.

Numerous calculations were performed with the CASTEP component in the Materials Studio [39,40], targeting precise simulations of the electronic structure and traits of monolayer GeSe. These calculations utilized the plane-wave pseudopotential technique, where the exchange-correlation energy was handled via the generalized gradient approximation (GGA) following the Perdew–Burke–Ernzerhof (PBE) approach. To ensure computational precision, an energy cutoff of 450 eV was set. The initial step of the study involved constructing the primitive cell model of a monolayer GeSe and performing detailed geometric optimization. The optimization results indicated that the lattice parameters of the resulting monolayer GeSe structure were a = 4.00 Å and b = 4.22 Å, aligning with existing literature reports [41,42], thus confirming the reliability of the computational model. Subsequently, by expanding the optimized primitive cell in-plane, a 3 × 3 × 1 supercell model was constructed, comprising 18 germanium (Ge) and 18 selenium (Se) atoms. To eliminate interlayer interactions that could arise during simulations, a vacuum layer of 20 Å was specifically implemented in the vertical direction of the monolayer GeSe model. In these calculations, the convergence criteria for atomic energies and forces were set to 1.0 × 10−5 eV/atom and 0.03 eV/Å, respectively, ensuring rigor in the computational process and precision in the results.

## 4. Summary

In summary, utilizing the physical vapor deposition (PVD) method, GeSe nanowires were successfully synthesized by heating GeSe powder at temperatures of 500 °C, 530 °C, 560 °C, 590 °C, and 620 °C. Through a series of optoelectronic performance tests, it was found that GeSe nanowires prepared at 560 °C exhibited outstanding performance under 780 nm light irradiation. These nanowires demonstrated high crystalline quality, uniform diameter, and significant dark current, photocurrent, and responsivity. These results underscore the significant potential of GeSe nanomaterials in the field of optoelectronic detection. Particularly in the visible light spectrum, the GeSe nanowires array displayed excellent light absorption capability and photo response, indicating its ability to rapidly convert external light signals into electrical output signals. These characteristics position GeSe nanomaterials with broad prospects in the field of optoelectronic detector devices.

## Data Availability

Data will be made available on request.
